# Bioassay-Guided Isolation and Active Compounds Identification of the AntiDiabetic Fractions of *Centaurea calcitrapa* Extract and the Predicted Interaction Mechanism

**DOI:** 10.3390/molecules30112394

**Published:** 2025-05-30

**Authors:** Hayder Mohammed Kadhim, Yasir M. Kadhim, Hayder Adnan Fawzi, Zaid M. Abdul Khalik, Ali Mohammed Jawad, Kamel Ghédira

**Affiliations:** 1 Pharmacognosy Laboratory, Faculty of Pharmacy, University of Monastir, Avicenna Street, Monastir 5000, Tunisia; kinaanhayder@gmail.com (H.M.K.); kamelghedira1@gmail.com (K.G.); 2 Department of Pharmaceutical Chemistry, College of Pharmacy, Al-Nahrain University, Baghdad 10006, Iraq; yr18121424@gmail.com; 3 Department of Clinical Pharmacy, College of Pharmacy, AlMustafa University, Baghdad 10064, Iraq; 4 Department of Pharmaceutics, Baghdad College of Medical Sciences, Baghdad 10047, Iraq; prof_x12@yahoo.com; 5 Scientific Research Authority, Environment and Water Research Center, Ministry of Science and Technology, Baghdad 10066, Iraq; alialtameme139@gmail.com

**Keywords:** *Centaurea calcitrapa*, aerial parts, antidiabetic, flavonoids, molecular docking

## Abstract

*Centaurea calcitrapa* is a well-known plant with antioxidant, anti-proliferative, and antimicrobial properties. The plant contains various phenolic compounds, flavonoids, and other bioactive molecules contributing to its medicinal properties. However, little is known about its antidiabetic activity. The study's purpose is the isolation and identification of active compounds of *C. calcitrapa* aerial parts in diabetic rats induced by streptozotocin. The ethyl acetate extract (E2) was separated into eight subfractions by column chromatography. The subfractions were evaluated for their antidiabetic activity using diabetic-induced rats. The most active subtraction was purified, and the active compounds were identified using UV spectrophotometry, Fourier Transform Infrared Spectroscopy, Mass spectrophotometry, and HPLC. Subfraction E2-VIII showed the most effective reduction in blood glucose levels, comparable to metformin. In HPLC analysis, subfraction E2-VIII showed three main compounds: nepetin, kaempferide, and Luteolin. The nepetin flavonoid was examined using molecular docking, and it showed a high affinity to α-amylase. In conclusion, the aerial parts of *C. calcitrapa* extract and isolated compounds especially nepetin present promising antidiabetic agents this is probably mediated by its strong antioxidants and α-amylase inhibitory effect.

## 1. Introduction

Diabetes mellitus (DM) is a chronic metabolic disorder characterized by persistent elevated blood glucose levels and alterations in insulin levels or action. Type 2 diabetes mellitus (T2DM) is the predominant form [1]. T2DM has a considerable burden on the healthcare system; in 2019, the International Diabetes Federation (IDF) reported that diabetes resulted in 4.2 million fatalities, with 463 million people aged 20 to 79 years living with the condition, a figure projected to increase to 700 million by 2045. The incidence and prevalence of T2DM differ by geographical region, with over 80% of patients residing in low- to middle-income nations presenting further obstacles to effective treatment [2].

The pathophysiology of T2DM is attributed to a combination of two principal factors: impaired insulin production by pancreatic β-cells and the inadequate responsiveness of insulin-sensitive tissues to insulin [2], in which a disruption in the feedback mechanisms between insulin activity and secretion leads to elevated blood glucose levels [3,4]. In case of β-cell malfunction, insulin production diminishes, constraining the body’s ability to regulate physiological glucose levels. Meanwhile, insulin resistance (IR) leads to heightened glucose synthesis in the liver and diminished glucose absorption in muscle, liver, and adipose tissue. Although both mechanisms occur early in pathogenesis and contribute to disease progression, β-cell dysfunction is typically more pronounced than IR. Nonetheless, the coexistence of β-cell malfunction and insulin resistance exacerbates hyperglycemia, facilitating the advancement of T2DM [5,6,7].

The persistent and extensive elevation of reactive oxygen species (ROS) plays a crucial role in the pathophysiology of T2DM and IR. A pro-oxidant environment results in mitochondrial malfunction, endoplasmic reticulum stress, NADPH oxidase (NOX) activation, and superoxide formation. The elevation of ROS production stimulates the five principal pathways implicated in the pathogenesis of diabetes complications: augmentation of the polyol pathway, heightened formation of advanced glycation end-products (AGEs), increased expression of AGEs receptors and their activating ligands, activation of protein kinase C (PKC) isoforms, and hyperactivity of the hexosamine pathway [8,9]. Increased intracellular ROS via these pathways leads to impaired angiogenesis in response to ischemia, activates many proinflammatory pathways, and induces constant epigenetic modifications that sustain the expression of proinflammatory genes even when hyperglycemia is resolved [10].

Despite the availability of various therapeutic options to treat T2DM, it still lacks a definitive cure. Consequently, scientists are actively exploring multiple methods to treat and prevent diabetes. One of the methods used is traditional and complementary medicine [11]. The World Health Organization (WHO) has defined traditional medicine, stating that it encompasses the comprehensive understanding of health practices and abilities rooted in indigenous beliefs and experiences. Complementary medicine, on the other hand, refers to a range of health practices that are not considered part of a particular country’s traditional medicine [11].

The *Asteraceae* family is among the largest flowering plant families, comprising approximately 20,000 species. The *Centaurea* genus is a prominent group within this family, comprising between 250 and 700 species. These species are herbaceous annuals, biennials, and perennials, and are widely distributed worldwide, especially in the Mediterranean region, Western Asia, and the Americas. The *Asteraceae* family consists of four main subfamilies, three of which are present in Iraq [12,13]. *Centaurea* species have been utilized for medicinal use for centuries, including antibacterial, anti-inflammatory, antipyretic, antirheumatic, antidiarrheal, and cytotoxic properties. The leaves and shoots of *Stepposa Wagenitz*., *C. urvillei* DC. spp., *C. triumfettii* All, *C. calcitrapa* L., and *C. pullata* L. have traditionally been included in the diet, either in their raw form or after processing. Additionally, certain species are utilized to produce drinks and tonics [14,15,16]. Phytochemical analyses of *Centaurea* species have revealed the existence of several natural chemicals that display diverse biological functions [14,17,18]. The genus *Centaurea* contains several active components, such as phenolic acids, sesquiterpene lactones, steroids, and flavonoids. These components contribute to the genus’s wide range of biological activities [19].

*Centaurea calcitrapa* (known as purple starthistle) is a biennial, herbaceous plant that typically reaches a height of 60 cm. This plant is traditionally used to manage several diseases, including ophthalmic disorders, as well as antipyretic, hepatic, gastrointestinal, and dermatological diseases [20,21]. Prior studies on its extracts demonstrated promising potential for biological action. The aqueous and methanol (MeOH) extracts demonstrated potent antioxidant activity. Additionally, the MeOH extract has shown considerable cytotoxic action against HeLa (human cervical cancer) and Vero (epithelial cells of African green monkey kidney) cell lines [22]. Phytochemical investigations have identified sterols, sesquiterpene lactones, lignans, bisabolenes, triterpenoids, and flavonoids as components of *C. calcitrapa* extracts [23,24,25]. The MeOH extract showed an antidiabetic effect attributed to its α-glucosidase inhibitory effect [26].

The molecular docking method is a bioinformatics model that examines protein-ligand interactions at the atomic scale. This interaction resembles the lock-and-key approach, utilized to identify target structures for protein active sites and clarify the possible mechanism of action. Conversely, ligands can associate with proteins via several interactions, namely hydrogen bonds, hydrophobic interactions, van der Waals forces, and salt bridges, and are defined by their binding affinity [27,28].

The current study addressed several knowledge gaps regarding *C. calcitrapa* as an antidiabetic treatment. First, to elucidate if *C. calcitrapa* works as an antidiabetic agent, the specific bioactive compounds in *C. calcitrapa* that are responsible for its antidiabetic effects, the mechanism by which the plant’s compounds lower blood glucose levels, and assess the safety of *C. calcitrapa*. In the current study, we undertook bioassay-guided isolation and active compound identification in diabetic rats induced by streptozotocin. Furthermore, a molecular docking study will be performed for the most active compound isolated.

## 2. Results

### 2.1. Centaurea calcitrapa Extracts Ameliorate Renal, Hepatic, and Oxidative Stress Changes Induced by Streptozotocin

**Figure 1:**
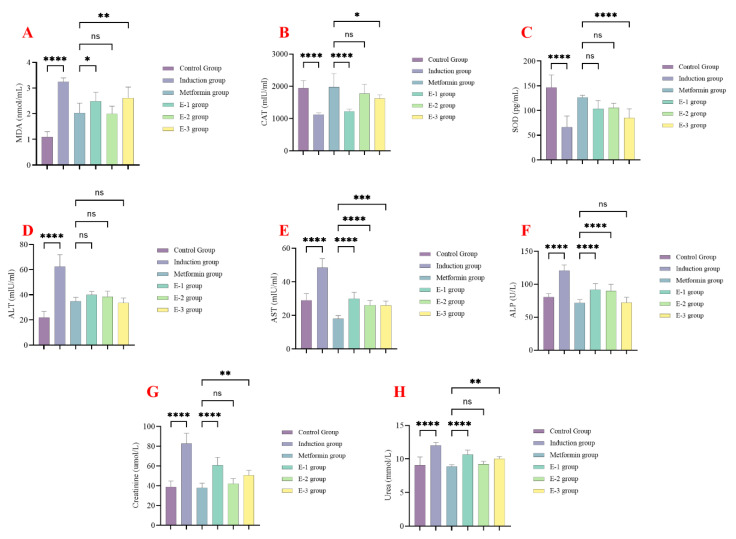
*Centaurea calcitrapa* extracts affect oxidative stress, liver enzymes, and kidney function markers. (**A**) MDA levels, (**B**) catalase levels, (**C**) SOD levels, (**D**) ALT levels, (**E**) AST levels, (**F**) ALP levels, (**G**) creatinine levels, and (**H**) urea levels. * Indicate *p*-value < 0.05, ** indicate *p*-value < 0.01, *** indicate *p*-value < 0.001, **** indicate *p*-value < 0.0001, ^ns^ indicate *p*-value > 0.05.

At the end of the experimental phase in the first experiment, serum levels of various biomarkers were assessed to examine the effect of *C. calcitrapa* extracts on liver function, kidney function, and oxidative stress changes induced by streptozocin. All extracts showed a significant reduction in the levels of MDA and CAT activity compared to the induction group and a significant increase in the levels of SOD compared to the induction group (Figure 1A–C). Ethyl acetate extract (E-2) of *C. calcitrapa* showed the best ameliorative effect on oxidative stress, showing no significant differences compared to the metformin group (Figure 1A–C).

All extracts significantly reduced ALT, AST, and ALP levels compared to the induction group, with comparable levels seen in the metformin group (Figure 1D–F). All extracts significantly reduced serum urea and creatinine compared to the induction group (Figure 1G,H). Extract E-2 demonstrated the most significant reduction in serum urea and creatinine compared to the metformin group. These findings collectively indicate that extract E-2 exhibited the best safety profile among the extracts.

### 2.2. Antidiabetic Properties of C. calcitrapa Extracts on Diabetic Rats Induced by Streptozotocin

**Figure 2:**
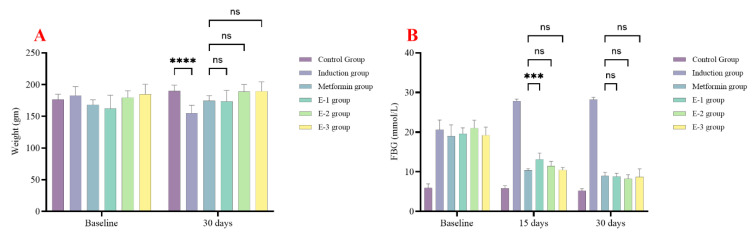
*Centaurea calcitrapa* extracts affect rat weight and glycemic control. (**A**) rat weight, (**B**) FBG at baseline, after 15 days, and 30 days. *** indicate *p*-value < 0.001, **** indicate *p*-value < 0.0001, ^ns^ indicate *p*-value > 0.05.

In the first experiment, rats were followed up after 15 and 30 days to examine changes in fasting blood glucose (FBG) and body weight. After 30 days, all rats in the extract groups showed no significant difference in body weight compared to the metformin group (Figure 2A).

Regarding glycemic control, after 15 days, only extracts E-2 and E-3 showed no significant difference compared to the metformin group. However, after 30 days, all extracts demonstrated no significant difference compared to the metformin group. At all times (15 and 30 days), all extracts showed significantly lower FBG levels compared to the induction group, as seen in Figure 2B.

As a result, since E-2 extracts showed the best safety profile and glycemic control, E-2 extract was selected for further bioassay.

### 2.3. Antidiabetic Properties of E-2 Subfractions on Diabetic Rats Induced by Streptozotocin

**Figure 3:**
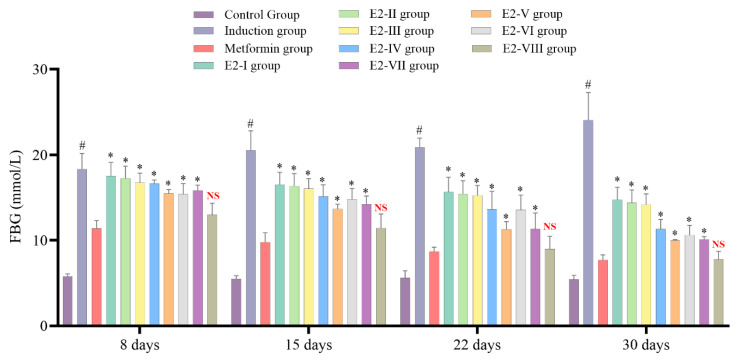
*Centaurea calcitrapa* subfraction effect on glycemic control after 8, 15, 22, and 30 days. * Indicates significant difference (*p*-value < 0.05) compared to the Metformin group, ^#^ Indicates significant difference (*p*-value < 0.05) compared to the Control group, and ^NS^ indicates *p*-value > 0.05.

After selecting the E-2 extract, it was further isolated by column chromatography, yielding eight subfractions. These subfractions were subjected to animal experimentation to determine the best antidiabetic properties. After 8, 15, 22, and 30 days, the E-2VIII subfraction showed no significant difference in FBG levels compared to the metformin group, which indicates that this subfraction contains most of the antidiabetic activity of *C. calcitrapa,* as seen in Figure 3. Subsequently, subfraction E-2VIII underwent purification and identification of its active phytochemical components.

### 2.4. Purification of E2-VIII Subfraction by HPLC

**Figure 4:**
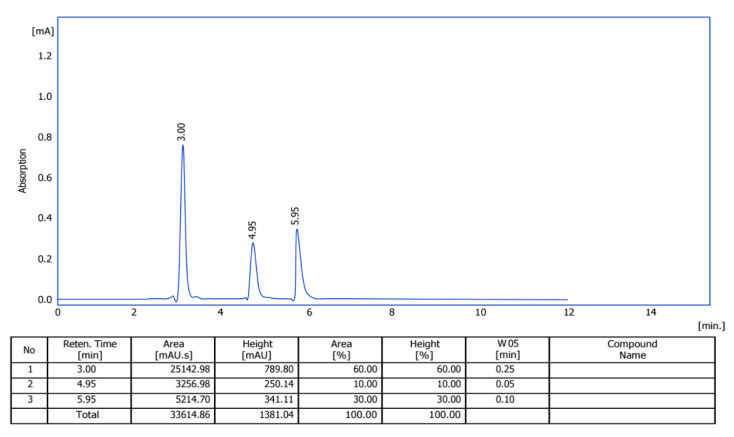
HPLC diagram of subfraction E2-VIII.

The HPLC purification of E2-VIII identified three compounds: compound **P-1** showed a peak at 3.0 min, compound **P-2** showed a peak at 4.95 min, and compound **P-3** showed a peak at 5.95 min, as shown by the three curves in Figure 4. Furthermore, we utilized HPLC to separate these three compounds for further analysis to elucidate their chemical structures.

### 2.5. Calibration of Nepetin, Kaempferide, and Luteolin Compounds by HPLC

See supplementary Material (Figure S1).

### 2.6. Structural Analysis of E2-VIII Subfractions

The HPLC chromatography’s separation technique separated E2-VIII into three portions; these portions undergo UV spectroscopy, Fourier-transform infrared spectroscopy (FT-IR), and mass spectrometry (MS), elucidating their chemical structure, then confirm it by comparing each one with HPLC with 10 standard compounds (Scutellarin, Luteolin, Nepetin, Apigenin, Kaempferol, Chryseriol, Jaceidin, Kaempferide, Eupatolin, and Centaureidin), as seen in Table 1.

**Figure 5:**
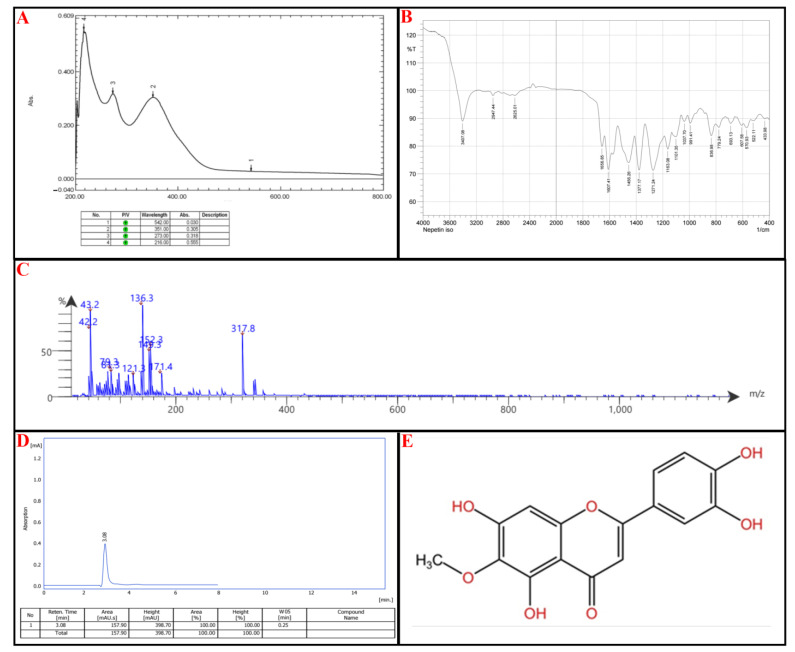
Identification of subfraction E2-VIII portion **P-1**, (**A**) UV spectrophotometry, (**B**) FT-IR, (**C**) MS-spectrometry, (**D**) HPLC, (**E**) chemical structure.

Compound I (**P-1**): UV-Vis Spectroscopy (273 nm and 351 nm): These absorbance wavelengths suggest the presence of a benzoyl peak 220–280 nm and a cinnamoyl peak 300–400 nm reference (Figure 5A).

FTIR Analysis: 3407 cm^−1^: Indicates the presence of an O-H stretch, from hydroxyl groups. Notably, 2947 cm^−1^: Suggests aromatic C-H stretching. Notably, 1657 cm^−1^ and 1607 cm^−1^: Strong evidence of C=O (carbonyl) and C=C (aromatic) stretches. Notably, 1456 cm^−1^ and 1377 cm^−1^: Points to C-H bending vibrations, characteristic of methyl (-CH₃) or methylene (-CH₂-) groups. Notably, 1271–1037 cm^−1^: Suggests the presence of C-O stretching (Figure 5B).

Mass Spectrometry (MS) Analysis: Parent Peak: 317.8 *m*/*z*: Indicates the compound’s molecular weight (M + H + 1), suggesting a relatively large structure. Notably, 316.8 (M + 1) and the molecular fragments 171.4; [M + H-C_9_H_6_O_2_] 149.3; M + H-C_7_H_4_O_5_, 136.3 (Figure 5C).

Based on the spectral data and the listed flavonoids’ structural characteristics, the most likely candidate is Nepetin or Jaceidin. The UV-Vis absorption suggests a flavonoid backbone with extended conjugation, likely a methoxylated flavone; Nepetin and Jaceidin exhibit similar UV profiles due to their conjugated systems. Based on FTIR analysis: 3407 cm^−1^ (O-H stretch): Present in flavonoids. Notably, 1657 cm^−1^ and 1607 cm^−1^ (C=O and C=C stretches): Strong indicators of flavone structures. Notably, 1271–1037 cm^−1^ (C-O stretches): Suggests ether or ester functionalities common in methoxylated flavones. Based on MS analysis, this molecular weight aligns closely with Nepetin (MW: 316.3 g/mol) and Jaceidin (MW: 330.3 g/mol). Fragmentation patterns (171.4, 152.3, 152.3, 149.3, 136.3, etc.) suggest a breakdown of a flavone core with methoxy substitutions.

The final step relied on HPLC; the isolated compound peaks at a retention time (Rt) of 3.08 min, comparable to the standard nepetin with an Rt of 3.08 min, as seen in Figure 5D. The chemical structure of nepetin, according to the International Union of Pure and Applied Chemistry (IUPAC), is 2-(3,4-dihydroxyphenyl)-5,7-dihydroxy-6-methoxy-4H-1-benzopyran-4-one, which is illustrated in Figure 5E (for reference standard of each analytical procedure, see Supplementary Material Figure S2).

**Figure 6:**
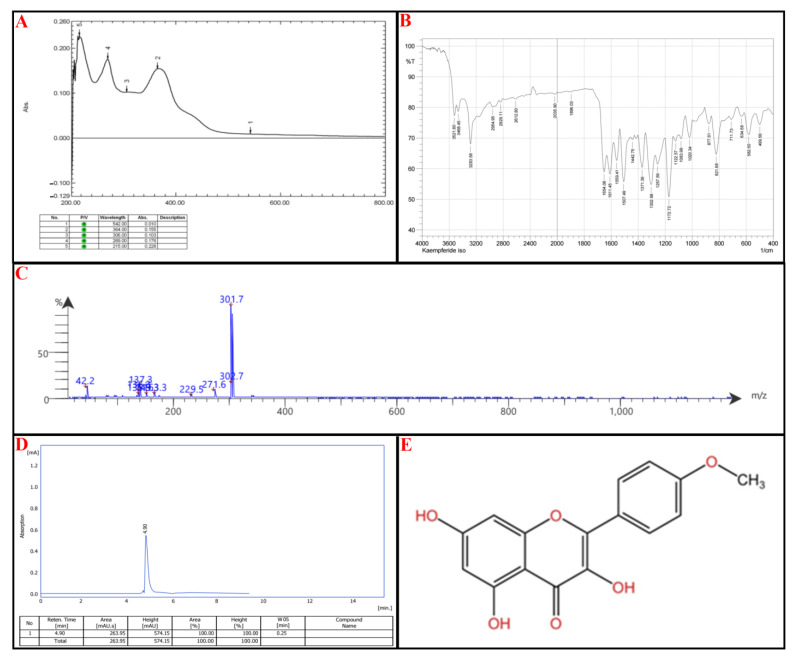
Identification of subfraction E2-VIII portion **P-2**, (**A**) UV spectrophotometry, (**B**) FT-IR, (**C**) MS-spectrometry, (**D**) HPLC, (**E**) chemical structure.

Compound II: UV-Vis Spectroscopy (269 nm and 364 nm): These absorbance wavelengths suggest the presence of a benzoyl peak 220–280 nm and a cinnamoyl peak 300–400 nm reference (Figure 6A).

FTIR Analysis: 3522–3284 cm^−1^: Broad O-H stretches, characteristic of phenolic groups. Notably, 2955–2829 cm^−1^: C-H stretching, likely from aliphatic or aromatic systems. Notably, 2613 cm^−1^ and 2036 cm^−1^: Uncommon absorption bands—could suggest weak hydrogen bonding or interactions. Notably, 1654 cm^−1^, 1611 cm^−1^, 1559 cm^−1^: Strong C=O and C=C stretches, confirming flavonoid backbone with carbonyl functional groups. Notably, 1258–1020 cm^−1^: C-O stretching bands suggest the presence of ether or ester functionalities (Figure 6B).

Mass Spectrometry (MS) Analysis: Parent Ion: 302.7 *m*/*z*: Suggests a molecular weight near 302 g/mol, which fits certain flavonoids. Fragmentation Patterns: 301.7, 271.6: Consistent with sequential losses of hydroxyl groups. Notably, 229.5, 163.3, 149, 137.3: Breakdown of the flavone core. Notably, 42.2 *m*/*z*: Typical of small alkyl fragment losses (Figure 6C).

Kaempferide has characteristic UV absorptions near 260–370 nm, corresponding to *π→π* transitions* in its conjugated flavone structure. The presence of a methoxy (-OCH₃) group at position 4′ can shift absorption toward higher wavelengths, which matches 364 nm. FTIR Analysis: Key bands of Kaempferide include a broad O-H stretching (~3500 cm^−1^) → Present in Compound II (3522, 3468 cm^−1^). C=O stretching (1650–1620 cm^−1^) → Found in Compound II (1654, 1611 cm^−1^). C=C stretching (aromatic core, ~1600–1550 cm^−1^) → Present (1559, 1507 cm^−1^). C-O stretching (~1260–1030 cm^−1^) → Matches Compound II (1258–1020 cm^−1^).

The final step relied on HPLC; the isolated compound peaks at an Rt of 4.90 min, comparable to the standard kaempferide, which has an Rt of 4.98 min, as seen in Figure 6D. According to IUPAC, the chemical structure of kaempferide is 3,5,7-Trihydroxy-2-(4-methoxyphenyl)-4H-chromen-4-one, illustrated in Figure 6E (for reference standard of each analytical procedure, see Supplementary Material Figure S3).

**Figure 7:**
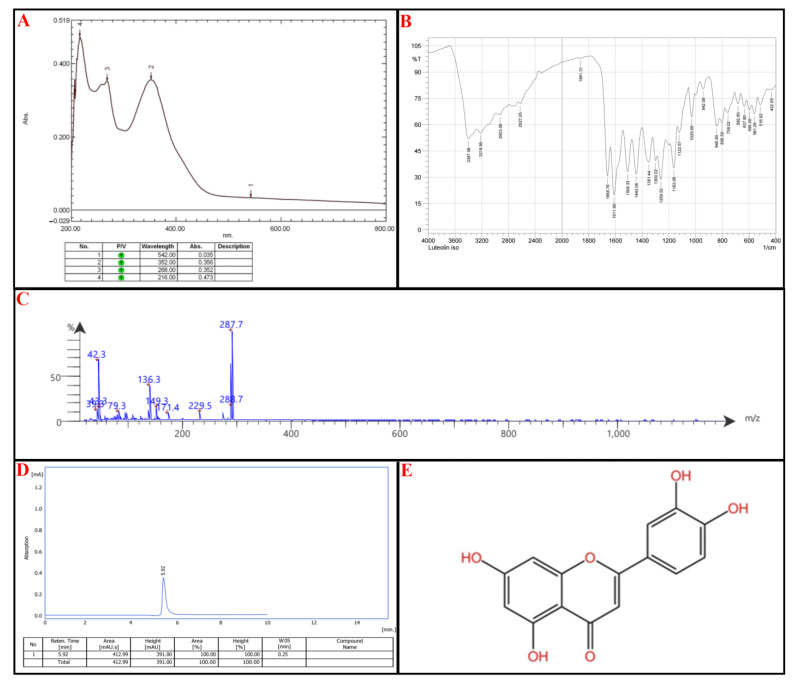
Identification of subfraction E2-VIII portion **P-3**, (**A**) UV spectrophotometry, (**B**) FT-IR, (**C**) MS-spectrometry, (**D**) HPLC, (**E**) chemical structure.

Compound III: UV-Vis Spectroscopy (268 nm and 352 nm): These absorbance wavelengths suggest the presence of a benzoyl peak 220–280 nm and a cinnamoyl peak 300–400 nm reference (Figure 7A).

FTIR Analysis: 3397 cm^−1^ and 3219 cm^−1^: Broad O-H stretching, confirming phenolic functional groups. Notably, 2923 cm^−1^: C-H stretching, typical of aromatic/aliphatic compounds. Notably, 1659 cm^−1^ and 1612 cm^−1^: C=O (carbonyl) and C=C (aromatic) stretching, consistent with flavone structures. Notably, 1260–1030 cm^−1^: C-O stretching, suggesting the presence of ether or ester functionalities (Figure 7B).

Mass Spectrometry (MS) Analysis: Parent Ion: 288.7 *m*/*z* → Suggests a molecular weight close to 288 g/mol, which aligns with flavonoids lacking methoxy groups. Fragmentation Patterns: 287.7, 229.5: Loss of hydroxyl groups. Notably, 171.4, 149.3, 136.3: Breakdown of the flavone core. Notably, 43.3, 42.3, 39.3: Alkyl fragment losses, confirming side-group modifications.

Among the listed flavonoids (Scutellarin, Luteolin, Nepetin, Apigenin, Kaempferol, Chrysoeriol, Jaceidin, Kaempferide, Eupatolin, Centaureidin), the best match is Luteolin (MW: 286 g/mol) → Its UV absorption, FTIR bands, and MS fragmentation pattern closely align. Luteolin has hydroxyl groups at key positions, which match the FTIR O-H stretch and mass fragmentation pattern. Other candidates, such as Kaempferol or Apigenin (MW: 286 g/mol), lack the necessary hydroxyl arrangement, making Luteolin the strongest match (Figure 7C).

HPLC was utilized to confirm the compound’s identity; the isolated compound peaks at an Rt of 5.92 min, comparable to the standard Luteolin with an Rt of 5.90 min, as seen in Figure 7D. According to IUPAC, the chemical structure of Luteolin is 2-(3,4-dihydroxyphenyl)-5,7-dihydroxy-4H-chromen-4-one, as illustrated in Figure 7E (for reference standard of each analytical procedure, see Supplementary Figure S4).

### 2.7. Molecular Docking

The antidiabetic mode of action targets α-amylase. Suppression of α-amylase enzyme activity diminishes glucose absorption in the intestines. It can efficiently postpone the release of glucose into the bloodstream, hence managing deteriorating health issues such as T2DM [30,31].

The nepetin flavonoid was thought to be the main contributor to the subfraction E2-VIII’s in vivo hypoglycemic effect. Therefore, in silico studies were conducted to predict their binding affinity with antidiabetic receptors (α-amylase). The computational approach we used involved docking the flavonoids to form a complex with α-amylase.

The protein-ligand complex is established via electrostatic forces at the binding interface, encompassing hydrogen bonds (from both side chains and backbones), salt bridges, and π-π stacking. Hydrogen bonding confers stability to protein molecules and specific protein-ligand interactions, making it crucial for the interactions of biological macromolecules.

**Figure 8:**
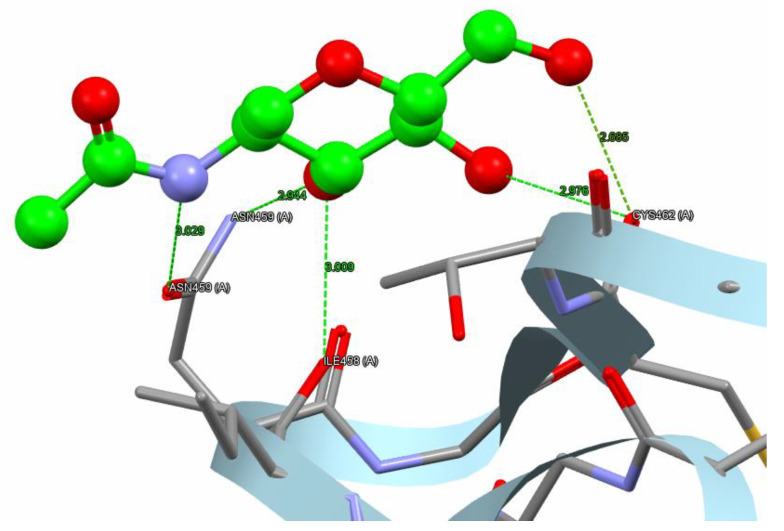
The reference compound hydrogen bonds and hydrophobic interaction with the α-amylase (PDB code: 1U2Y). The interaction between them via hydrogen bonds [ASN459, CYS462, and ILE458] is green, while brief contact is red. [The reference compound is administered in a ball-and-stick fashion, whereas amino acids are administered as capped sticks].

**Figure 9:**
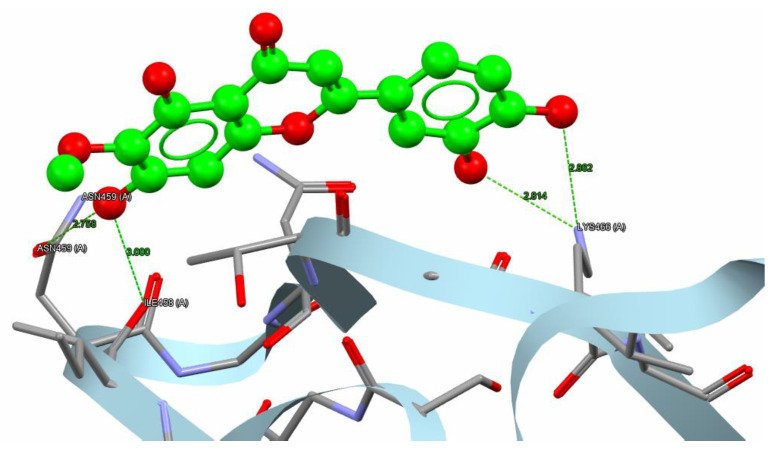
Nepetin hydrogen bonds besides hydrophobic interaction with α-amylase (PDB code: 1U2Y). The interaction between them via hydrogen bonds [ASN459, ILE458, and LYS 466] is in green, while brief contact is in red. [The nepetin compound is administered in a ball-and-stick fashion, whereas amino acids are administered as capped sticks].

The variation in binding energies of nepetin (44.66 Kcal/mol) indicates a superior binding affinity compared to the reference compound (31.96 Kcal/mol) for the α-amylase target. Nepetin formed four hydrogen bonds (2.758–3 Å) with three amino acids: ASN459, ILE458, and LYS466 in the α-amylase target. Simultaneously, 1U2Y formed five hydrogen bonds (2.685–3.029 Å) with three amino acids: ASN459, CYS462, and ILE458 in α-amylase, as illustrated in Figure 8 and Figure 9.

### 2.8. In Vitro α-Amylase Inhibitory Activity of Nepetin

**Figure 10:**
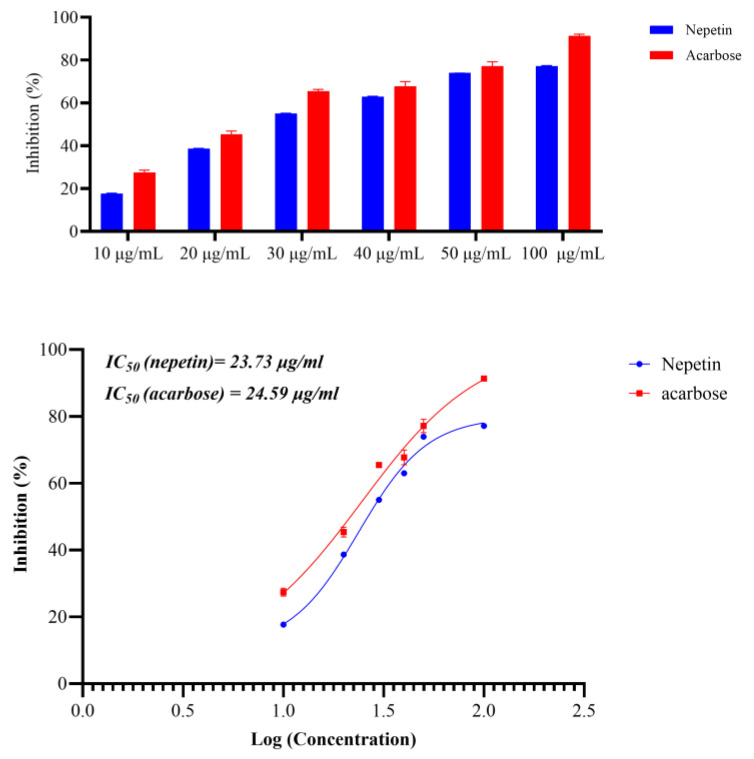
Inhibitory activity of nepetin and acarbose against α-amylase showing the percentage inhibition and IC_50_ (R^2^ of nepetin is 0.9933 and for acarbose is 0.9779, using log(inhibitor) vs. response-Variable slope (four parameters).

To confirm the antidiabetic action of nepetin, which was obtained in silico, we conducted an in vitro experiment to confirm the α-amylase inhibitory activity of nepetin utilizing Bernfeld’s technique, with Acarbose serving as the standard. The nepetin exhibited strong α-amylase inhibitory activity, comparable to acarbose, with the IC50 ± SE of 23.73 ± 0.2619 vs. 24.59 ± 0.5151 μg/mL for nepetin and acarbose, respectively, as seen in Figure 10.

## 3. Discussion

The present study examines the potential of *C. calcitrapa* extract and its subfractions as an antidiabetic agent; one subfraction showed potent antidiabetic activity and high safety (E2-extract). The E2 extract demonstrated no toxicity, as evidenced by the ALT, AST, and ALP levels, which are crucial indicators for liver toxicity assessment. Similarly, the E2 extract showed no renal toxicity, as confirmed by the urea and creatinine levels in rats’ serum. This safety profile of the E2 extract provides reassurance for its potential use as an antidiabetic agent. The E2 extract demonstrates significant antioxidant activity, as evidenced by the levels of MDA, catalase, and SOD. Since oxidative stress is considered an important factor in the pathogenesis of T2DM, diabetes is associated with a reduction in antioxidant levels due to protein glycation. In contrast, elevated MDA levels suggest increased lipid peroxidation and free radicals. Following treatment with the plant extract, the antioxidant enzyme levels were compared to those in rats treated with metformin, indicating the antioxidant activity of the *C. calcitrapa* extract.

Our findings agreed with a previous study examining the renoprotective effects of *Centaurea choulettiana* leaves in mice treated with cisplatin, in which the plant n-butanol extract reduced urea and creatinine levels. In addition, the n-butanol extract showed potent antioxidant activity, as evidenced by improvement in renal tissue levels of MDA, reduced glutathione (GSH), CAT, SOD, and myeloperoxidase (MPO) [32]. Another study that examined the n-butanol extract of the aerial part of *Centaurea tougourensis* showed hepatorenal protection in mice treated with streptozotocin, as evidenced by a reduction in the levels of ALT, AST, creatinine, and urea [33]. The aqueous extract of *Ephedra foeminea* showed hepatorenal protective effects and potent antioxidant activity in diabetic rats treated with streptozotocin, with a significant reduction in ALT, AST, ALP, bilirubin, urea, and creatinine. Additionally, it showed a reduction in the interleukin (IL)-1 and GSH levels, which is in agreement with the current study [34]. These findings indicate that the species of the genus *Centaurea*, including *C. calcitrapa*, protect against streptozotocin-induced diabetic changes.

Streptozocin-induced diabetes can be attributed to pancreatic beta-cell death, leading to insulin insufficiency [35]. Additionally, streptozotocin-induced diabetes leads to heightened proinflammatory status, a hyperlipidemic state, and a redox imbalance, all of which are hallmarks of diabetes mellitus [10,36]. In the current study, metformin showed successful antidiabetic activity in rats; metformin is recognized for its efficacy in reducing blood glucose levels in individuals with type 2 diabetes; this is mainly achieved by suppressing hepatic gluconeogenesis and enhancing peripheral insulin sensitivity [37,38].

The diabetes condition is linked to a widespread elevation in tissue oxidative stress, potentially demonstrated by alterations in the tissue antioxidant system [39]. Oxidative stress may lead to excessive generation of oxygen-free radical precursors and/or diminished efficacy of the antioxidant system. The emergence of oxygen-free radicals is linked to the auto-oxidation of glucose, disrupted glutathione metabolism, modifications in antioxidant enzymes, and the production of lipid peroxides [40]. Numerous research studies have established the correlation between oxidative stress and the pathophysiology of insulin resistance through the suppression of insulin signaling and dysregulation of adipocytokines [41]. Elevated ROS synthesis among T2DM patients is believed to initiate numerous unfavorable processes, including hexosamine pathways, the development of advanced glycation end-products (AGEs), and PKCβ1/2 activation [42]. Hyperglycemia may produce oxidative stress through multiple pathways, including glucose autoxidation, the polyol pathway, advanced glycation end-product (AGE) production, and PKCβ1/2 kinase activation. In T2DM patients, increased levels of free fatty acids, leptin, and other circulating variables may contribute to the overproduction of ROS [40].

In the present study, subfraction E2-VIII after 8, 15, 22, and 30 days showed a sustained and consistent glucose-lowering effect compared to metformin. The E2-VIII subfractions were purified to determine the active components, and three components were identified: nepetin, kaempferide, and Luteolin. All these flavonoids were previously reported in *C. calcitrapa*, including nepetin, kaempferide, and Luteolin [12,14]. Furthermore, Nepetin was examined using molecular docking, showing that nepetin has strong α-amylase inhibitory activity. Based on these findings, we performed an in vitro study to show nepetin α-amylase inhibitory activity; nepetin had strong α-amylase inhibitory activity comparable to the active standard (acarbose) (IC50 ± SE of 23.73 ± 0.2619 μg/mL). These findings indicate that nepetin is the most important constituent and shows antidiabetic activity mediated through inhibiting the α-amylase enzyme.

Nepetin has been documented to exhibit several biological functions, notably anti-inflammatory responses [43] and antidiabetic activity [44]. Recently, nepetin was reported to inhibit the catalytic activity of protein tyrosine phosphatases (PTPN)-1, PTPN2, and PTPN11 in vitro, indicating that nepetin acts as a multi-targeting inhibitor; furthermore, treatment of mature 3T3-L1 adipocytes with 20 μM nepetin stimulates glucose uptake through AMPK activation, which indicates nepetin activity as an antidiabetic agent through ameliorating IR [44]. Nepetin can hinder the degranulation and the production of leukotriene C4 and prostaglandin D2 in IgE/antigen (Ag) stimulated bone marrow-derived mast cells in mice. The IgE/Ag-mediated signaling pathway indicated that nepetin inhibited intracellular Ca^2+^ levels and activated PLCγ1 and cPLA2. Moreover, nepetin administration diminished prostaglandin D2 synthesis and inhibited cyclooxygenase-2 protein expression by obstructing the Akt and nuclear factor-κB signaling pathways [43]. Nepetin also inhibits the activity of IL-1β-induced IL-6, IL-8, and MCP-1 secretion and mRNA expression by repressing the activation of NF-κB and MAPKs [45]; other studies showed that nepetin possesses anti-inflammatory activity by attenuating NF-κB [46].

Kaempferide showed multiple biological activities, like antioxidant [47], anti-inflammation [48], anticancer [49], antihypertension [50], and improving glycolipid metabolism disorder [51]. As an antioxidant and anti-inflammatory agent, Kaempferide exerts its biological activity by inhibiting the TLR4/IκBα/NFκB signaling pathways [48]. Kaempferide improves glycolipid metabolism disorder by activating the PPARγ and its downstream signaling pathway [51]. PPARγ modulates glucose metabolism primarily by enhancing the sensitivity of peripheral tissues to insulin, promoting glucose consumption in muscle, and suppressing hepatic glycogen production [52,53]. PPARγ may facilitate insulin sensitivity through many mechanisms. PI3K is a crucial enzyme facilitating glucose entry into cells. Activated PPARγ may enhance the PI3K/AKT signaling pathway to improve insulin sensitivity [54]; Activated PPARγ can elevate glucose transporter-4 expression, augment glucose uptake, and ameliorate insulin resistance [55]; PI3K activation may expedite triglyceride decomposition in peripheral tissues, elevate its synthesis in adipose tissue, and suppress glucagon production [56]. These findings suggest that kaempferide may exert its antidiabetic activity via activation of PPARγ, leading to insulin sensitization.

Luteolin is utilized to address multiple medical conditions by modulating oxidative stress, inflammation, and dyslipidemia, and decelerating carbohydrate digestion and absorption through interaction with α-glucosidase [57,58]. Recent research demonstrated that Luteolin had strong antidiabetic benefits in streptozotocin-induced diabetic rats. Luteolin supplementation over three weeks markedly reduced hyperglycemia, HbA1c levels, hyperlipidemia, and inflammation and enhanced antioxidant enzyme activity [59]. Luteolin can regenerate pancreatic β-cells and secrete insulin due to its ability to stimulate the release of bound insulin from β-cells by inhibiting ATP-sensitive K^+^ channels [60].

Thus, the heightened antidiabetic activity shown by subfraction E2-VIII could be attributed to its individual component to enhance insulin levels, insulin sensitivity, and anti-inflammatory activity.

## 4. Materials and Methods

### 4.1. Chemicals, Reagents

The solvents (methanol, ethyl acetate, chloroform, n-hexane, and petroleum ether) were purchased from Central Drug House (P) Ltd., Delhi, India. HPLC analytical grade solvents (Water and methanol) were purchased from Loba Chemie; the lab is in Mumbai, India. Soxhlet apparatus (Adarsh Scientific Industry, Ambala Cantt, India), rotavapor R-100 (Buchi Labortechnik AG, Flawil, Switzerland), fertigfolien/pre-coated thin-layer chromatography sheet allugram Xtra sil G/uv 254, Layer: 0.20 mm with fluorescent indicator UV 254 (Düren, Germany), column chromatography comax column, Czech Republic, packed with silica gel (60 μm, Merck Co., Darmstadt, Germany).

Catalase (Cusabio, Wuhan, China), Alkaline Phosphatase (ALP) (Cusabio, China), Rat aspartate aminotransferase (AST) (Cusabio, China), alanine aminotransferase (ALT) (Cusabio, China), superoxide dismutase (SOD) (Cusabio, China), Malondialdehyde (MDA) (MyBioSource, UDA, San Diego, CA, USA), urea (Linear, Barcelona, Spain), creatinine (Linear, Spain)).

### 4.2. Plant Materials

Assistant professor Dr Abd Al-Moein authenticated the plant in the University of Kirkuk, College of Medicinal and Industrial Plants, Iraq, a collection of *C. calcitrapa* flowers, leaves, and stem (the whole aerial part at blooming season).

A voucher specimen was deposited in the scientific affairs of the University of Kirkuk, College of Medicinal and Industrial Plants, under the accession number (2597/40/7).

The aerial parts of the plant are dried in a well-ventilated place in the shade, away from sunlight [61], and collected from Al-Taji town in Baghdad (33.494606, 44.170728). The whole aerial plant part is ground by an industrial grinder to reduce the plant particle size [62].

### 4.3. Extraction

The ground plant materials (4.0 kg) were defatted using petroleum ether [63], followed by the continuous extraction method (Soxhlet) using three different solvents with increasing polarity: chloroform, ethyl acetate, and methanol for 4.5 h for each solvent [64]. The resulting extract solution was filtered and concentrated using a rotary evaporator (Rotavapor^®^ R-100, BÜCHI Labortechnik AG, Flawil, Switzerland).

These extracts were evaluated for their potential antioxidant, antidiabetic, kidney function, and hepatic function activities: chloroform extract (E1), ethyl acetate extract (E2), and methanol extract (E3) in the diabetic-induced model in rats. (see Supplementary Figure S5).

### 4.4. Column Chromatography

Column chromatography technology was used to separate the components of E2, the normal phase (silica gel 60 μm), and the mobile phase gradient chloroform-methanol (100:0 to 0:100 *v*/*v*). The eluent is collected (each 10 mL) and examined by thin-layer chromatography (TLC) on silica gel with fluorescent indicator 254 nm on aluminum cards (layer thickness 0.2 mm) using n-hexane: ethyl acetate (3:1) as eluent (*v*/*v*), combining similar parts [65].

Eight fractions were isolated: E2-I, E2-II, E2-III, E2-IV, E2-V, E2-VI, E2-VII, and E2-VIII. All were evaluated for their antidiabetic activity, and the most active fraction was subjected to identification and structure elucidation. Details are illustrated in Supplementary Figure S4.

### 4.5. Purification of E2-VIII Subfraction by High-Performance Liquid Chromatography (HPLC)

The chromatographic separation and purification of E2-VIII individual components were conducted using a Hypersil ODS-C18 column (250 mm × 4.6 mm, 5 μm) reverse-phase HPLC system (SYKAM, Eresing, Germany) coupled with a UV diode array detector, L-2200 autosampler, and L-2130 pump. Chromatographic data were processed by Clarity Chromatography data station software v6.0. The isocratic mobile phase consisted of Methanol: Water (50:50, *v*/*v*), eluted at a 1 mL/min flow rate. The detector’s wavelength was 260 nm, and the sample injection volume was 100 μL.

### 4.6. Identification of the Isolated Compounds

#### 4.6.1. UV Spectrometry

The UV spectrometric experiments were conducted on a Shimadzu-1600, Japan. The scan range was 200 to 800 nm, the spectral band was 2.0 nm, and the spectral resolution was 0.1 nm. The light source was 340.8 nm. The samples analyzed were diluted in methanol into quartz cuvettes.

#### 4.6.2. Fourier-Transform Infrared Spectroscopy (FTIR)

The FTIR spectrometric experiments were carried out on a Shimadzu FTIR Prestige 21, Tokyo, Japan, with a scan range of 4000 to 400 cm^−1^, on KBr disks, spectral resolution 2 cm^−1^, He-Ne laser.

#### 4.6.3. Mass Spectrophotometry (MS)

The analysis was performed using Advion expression, New York, NY, USA. Ion Source: APCI, Polarity: Positive ion, *m*/*z* Range 10 to 1200, Acquisition Speed 10,000 *m*/*z* units/s, stability ± 0.1 *m*/*z* units over 12 h period (65–75 °F (18–24 °C) operating temperature), Polarity Switching Speed 50 Ms, Dynamic Range 4–5 orders of magnitude, Gas Supply 60 psi, >98% pure Nitrogen, Gas Consumption < 10 L/min, Pirani pressure 7.21 × 10^−3^ mbar, Turbo speed 99.6%, Capillary temperature 255 °C, Source gas temperature 354 °C, Transfer line temperature 0 °C, Capillary voltage 192.4 volts, Source voltage 76.5 volts, Extraction electrode 9.44 volts, Esi voltage 3.69 KV, Apci current 5.34 μamps, Hexapole bias 8.42 volts, Pole bias −5.11 volts, Hexapole RF 200.2 volts, Rectified RF 4.12 volts, Dc_1_ −97.29 volts, Dc_2_ 104.52 volts, Detector −1.15 KV, and Dynode −10.36 KV.

#### 4.6.4. Calibration Curve

Nepetin, kaempferide, and luteolin standard compounds were dissolved in MeOH (1 mg/mL). All samples were filtered through a syringe filter before analysis. Standard solutions were serially diluted to prepare different concentrations (3, 5, 7, and 9 μg/mL). A calibration curve for each standard was constructed by plotting concentration (*x*, ppm) versus peak area (*y*). Linearity was assessed from the correlation coefficient value (*r*^2^) [66]. Details are illustrated in Supplementary Figure S5.

### 4.7. In Vitro α-Amylase Inhibitory Assay

The α-amylase inhibitory activity of nepetin was assessed using Bernfeld’s technique, with Acarbose serving as the standard [67]. A series of nepetin and acarbose with concentrations of 10, 20, 30, 40, 50, and 100 μg/mL was made and permitted to react with α-amylase and 2 mM phosphate buffer (pH 6.9). Following a 20-minute incubation, 0.1 mL of 1% starch solution was included in the reaction mixture. A similar method was executed for control samples devoid of the enzyme. Subsequently, 0.5 mL of dinitro salicylic acid reagent was introduced to both the control and test samples and maintained in a boiling water bath for 5 min. The absorbance was subsequently measured at 405 nm utilizing a spectrophotometer, and the percentage inhibition was computed using the formula [26,68]:

$$Inhibition\;\left(\%\right)=\frac{A_{C}-A_{T}}{A_{C}}\times 100\%$$

*A_C_* indicates the absorbance of the control (containing all reagents except the test solution), and *A_T_* indicates the absorbance of the tested solution.

The half-maximal inhibitory concentration (IC50) values were identified by applying nonlinear regression to fit inhibition parameters with standard log inhibitors against 4P-response models [69].

### 4.8. Experimental Design and Settings

Sprague-Dawley albino male rats aged 12 to 16 weeks, weighing 140–220 grams, were obtained from a biotechnology research center at Tikrit University. All care for animals and scientific experiments were conducted in strict compliance with regulations established by the animal ethics committee at the Al-Mustafa University College animal facility in Baghdad, Iraq (following AVMA guideline 2020 [70]).

Animals were adjusted for seven days in laboratory settings prior to the commencement of the assessments. Water and standard food pellets (Elazig Food Company, Elazig, Turkey) were provided ad libitum in the ventilated room. Before and during the experimental period, all rats were assessed for their health status, including food and water intake. A 12 h light-dark period, ambient temperature of 18–22 °C, and 40% humidity. The room was adequately aired with entirely fresh air. The authors adhered to the ARRIVE 2.0 criteria [71].

The study was conducted at Al-Mustafa University College’s animal house between 5 January 2022, and 23 April 2024.

The experimental phase includes two animal studies. The initial study aimed to determine the antidiabetic activity of plant extracts, and then the extract with the highest hepatorenal safety and best antidiabetic activity would be further investigated; the second experimental study aimed to identify the active compound with the highest antidiabetic activity.

#### 4.8.1. Diabetic Induction

The Sprague-Dawley albino male rats were acclimated and subjected to an overnight fast. Subsequently, they were administered a single intraperitoneal injection of a freshly prepared solution of streptozotocin (STZ) (60 mg/kg body weight) in a 0.1 M cold citrate buffer (pH 4.5). During the induction process, the rats were given a 5% glucose solution overnight to prevent severe low blood sugar caused by the excessive release of insulin caused by the administration of STZ. The rats were categorized as diabetic if their blood glucose levels surpassed 13.9 mmol/L 72 h following the treatment with STZ. The administration of investigational treatment commenced on the fourth day following the STZ injection. Rats in the control group that did not have diabetes induced were exposed to the identical procedure; instead of receiving STZ, they were given intraperitoneal injections of a solution containing 0.9% saline [38,72].

#### 4.8.2. Special Considerations to Minimize the Suffering and Distress of Animals

Every attempt was made to reduce suffering and the number of animals participating in the tests. The animal was observed immediately following the STZ injection, approximately 10 min later, and the subsequent day. In the event of bleeding, gauze was applied, and pressure was exerted. After the hemorrhage ceased, the area was sanitized with gauze and water. In instances of peritonitis, internal organ laceration, and/or infection, a veterinarian was consulted to evaluate the animal’s suitability for further participation in the experiment [73,74].

#### 4.8.3. Plant Extracts Experimental Design

**Figure 11:**
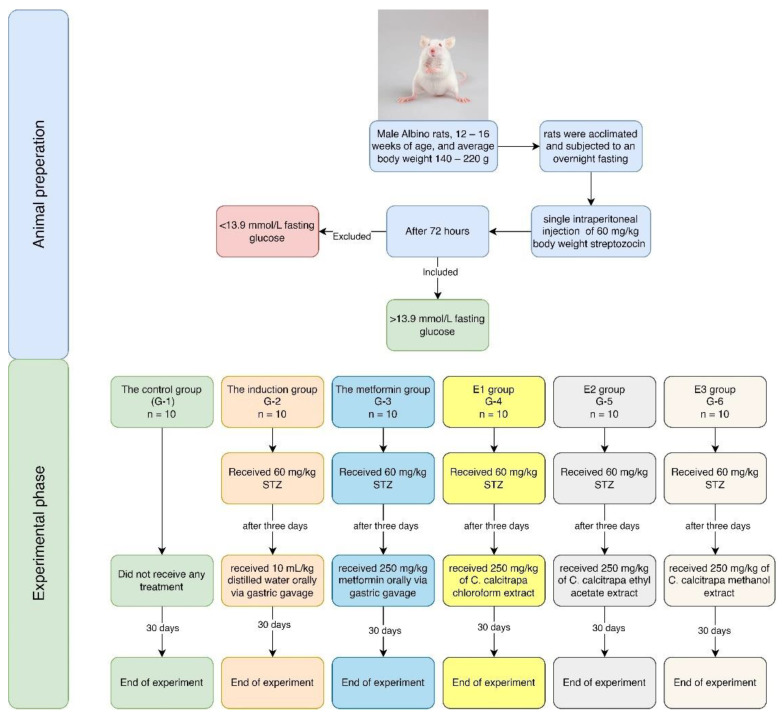
Flow chart of the experiment.

The initial study included 60 rats divided into six groups (n = 10 for each group). The experiment continued for 30 days with daily administration of metformin and the investigated extracts; at the end of the experiment, all animals survived, as illustrated in Table 2 and Figure 11.

#### 4.8.4. *C. calcitrapa* Ethyl Acetate Extract (E2) Fractions Experimental Design

The second experiment involved 88 rats divided into 11 groups (n = 8 for each group). It continued for 30 days with daily administration of metformin and the investigated subfractions. As illustrated in Table 3, all animals survived at the end of the experiment.

### 4.9. Clinical and Laboratory Assessment

#### 4.9.1. Body Weight Monitoring

Rats’ body weights were observed at the experiment’s start and end.

#### 4.9.2. Monitoring the Blood Glucose Level

The blood glucose level was measured by scratching the tail vein using the Accu-Chek Performa blood glucometer (Roche, Mannheim, Germany) [38]. The fasting blood glucose (FBG) level measurement was taken in the first experiment on the 1st, 15th, and 30th day of the experimental work. In the second experiment, the blood glucose level measurement was taken on the 1st, 8th, 15th, 22nd, and 30th day of the experimental work.

#### 4.9.3. Biochemical Analysis (Conducted on Stage 1)

Blood was collected in a clot activator tube, and the serum was separated by a centrifuge at 3000 rpm for 10 min. The serum was placed at −20 °C until analyses were performed.

Biomarkers of serum samples were assessed using the enzyme-linked immunosorbent assay (ELISA) technique. Frozen specimens were allowed to thaw at ambient temperature, and the biomarkers necessitating evaluation for the study comprised ALP (Cat# CSB E11865r), catalase (Cat# CSB E13439r), AST (Cat# CSB E13023r), ALT (Cat# CSB E13024r), SOD (CSB EL022397RA), MDA (Cat# MBS263626).

Creatinine (Ref# 1123005) was assessed using the kinetic colorimetric method based on picrate reaction (Jaffe) [78], and urea (Ref# 1158005) was assessed using an enzymatic method in which urea is hydrolyzed by urease to ammonia and carbon dioxide. The ammonia is converted to glutamate by glutamate dehydrogenase in the presence of NADH and oxoglutarate [79,80].

### 4.10. In Silico Molecular Docking Studies

The structure of nepetin had been drawn in ChemDraw Professional software (v. 16.0). The energy of each molecule was minimized using licensed CCDC genetic optimization for ligand docking (GOLD) Hermes 2021.2.0 (Build 327809) was used to achieve the molecular docking studies for the compounds and to envisage: the protein, ligands, interactions of hydrogen bonding, short contacts, and length of bonds calculation, to carry out the docking simulation. Protein molecules of α-amylase (PDB: 1U2Y) were retrieved from the protein data bank. The receptors were removed, and only polar hydrogen charges were added to the water molecules. GOLD was compiled and run under Windows 10.0 Professional operating system.

### 4.11. Ethical Considerations

The study was approved by the research ethical committee of Al-Mustafa University College (ID: AP023, date: 20 November 2021).

### 4.12. Statistical Analysis and Sample Size Calculation

Program G.Power3.1 was utilized for sample size calculation; post hoc sample size was conducted with an effect size of 0.5, power 80%, and an alpha level of 0.05, F-family tests with a total sample size of 60 for each group of 10 animals for the first experiment, while for the second experiment, an effect size of 0.47, power 80%, and an alpha level of 0.05, F-family tests with a total sample size of 88 for each group of 8 animals [81,82]. Random numbers were utilized to create groups in an Excel spreadsheet. The rats were placed in labeled cages and assigned tail tags to reduce confusion [83].

The Anderson-Darlin normality test was performed, and all variables followed a normal distribution. Ordinary One-way ANOVA with post hoc Tukey test was used to assess the effect of renal, hepatic, and oxidative stress markers. In contrast, two-way repeated measures ANOVA with post hoc Šídák’s multiple comparisons test was used to assess the difference in FBG and body weight [84]. The significance level was defined by *p*-value ≤ 0.05 (alpha level). All analyses used GraphPad Prism version 10.2.0 for Windows, GraphPad Software, and Boston, MA, USA.

## 5. Conclusions

This study underscores the potential of *C. calcitrapa* extract, particularly the E2 subfraction, as a promising antidiabetic agent with a strong safety profile. The absence of hepatotoxicity and renal toxicity, combined with its potent antioxidant activity, supports its viability for therapeutic applications. The extract not only demonstrated significant glucose-lowering effects but also provided antioxidant protection against oxidative stress—a major contributor to T2DM pathogenesis. The identified bioactive compounds, including nepetin, kaempferide, and luteolin, exhibit multifaceted biological activities that enhance insulin sensitivity, reduce inflammation, and promote antioxidant defenses. The in silico and in vitro studies indicate that the main compound shows activity against α-amylase.

Future research should focus on elucidating the precise molecular mechanisms through which these flavonoids exert their antidiabetic effects. Investigating their potential for synergistic interactions or formulation into optimized drug delivery systems may enhance their efficacy and stability. Moreover, expanding preclinical studies to include long-term toxicity assessments and evaluating their impact on insulin signaling pathways will be crucial for translating these findings into clinical applications. Given the promising results observed, the *C. calcitrapa* extract could pave the way for novel phytotherapeutic interventions in diabetes management, offering a natural and effective alternative to conventional therapies.

## Figures and Tables

**Table 1:** Structural analysis of E2-VIII subfractions.

Analysis	P-I	P-II	P-III
**Benzoyl peak**			
**Cinnamoyl peak** [29]	273	269	268
**Extended conjugation**	351	364	352
**FT-IR analysis**			
**O-H stretch**	3407	3522–3284	3397 and 3219
**Aromatic C-H stretching**	2947	2964–2829	2923
**C=O (carbonyl) and C=C (aromatic) stretches**	1656 and 1607	1654–1611	1659 and 1612
**C-H bending vibrations**	1456 and 1377	1371	1443 and 1351
**C-O stretching**	1271–1037	1258–1020	1260–1030
**MS analysis**			
**M + H + 1**	317.8	302.7	288.7
**M + H**	316.8	301.7	287.7
**M + H-CH_2_O**		271.6	
**M + H-C_3_H_6_O**			229.5
**M + H-C_9_H_6_O_2_**	171.4		171.4
**M + H-C_7_H_4_O_5_**	149.3		
**C_7_H_4_O_3_**	136.3		136.3
**C_7_H_5_O_3_**		137.3	
**C_8_H_9_O**	121.3		
**C_2_H_2_O**	42.2	42.2	42.3

**Table 2:** Experimental design of plant extracts animal study.

Group	Details	Procedure
**G-1**	The control group	Did not receive any treatment
**G-2**	The induction group	Received 60 mg/kg STZ and, after three days, received 10 mL/kg distilled water orally via gastric gavage daily for 30 days [75]
**G-3**	The metformin group	Received 60 mg/kg STZ and, after three days, received 250 mg/kg metformin orally via gastric gavage daily for 30 days [72,76,77].
**G-4**	*C. calcitrapa* chloroform extract (E1) group	Received 60 mg/kg STZ and, after three days, received 250 mg/kg of *C. calcitrapa* chloroform extract daily for 30 days.
**G-5**	*C calcitrapa* ethyl acetate extract (E2) group	Received 60 mg/kg STZ and, after three days, received 250 mg/kg of *C. calcitrapa* ethyl acetate extract daily for 30 days.
**G-6**	*C calcitrapa* methanol extract (E3) group	Received 60 mg/kg STZ and, after three days, received 250 mg/kg of *C. calcitrapa* methanol extract daily for 30 days.

**Table 3:** Experimental design of the plant extract fractions animal study.

Group	Details	Procedure
**S-1**	The control group	Did not receive any treatment
**S-2**	The induction group	Received 60 mg/kg STZ and, after three days, received 10 mL/kg distilled water orally via gastric gavage daily for 30 days [75]
**S-3**	The metformin group	Received 60 mg/kg STZ and, after three days, received 250 mg/kg metformin orally via gastric gavage daily for 30 days [72,76,77]
**S-4**	*C calcitrapa* ethyl acetate extract fraction I (E2-I) group	Received 60 mg/kg STZ and, after three days, received 100 mg/kg (E2-I) fraction orally via gastric gavage daily for 30 days
**S-5**	*C calcitrapa* ethyl acetate extract fraction II (E2-II) group	Received 60 mg/kg STZ and, after three days, received 100 mg/kg (E2-II) fraction orally via gastric gavage daily for 30 days
**S-6**	*C calcitrapa* ethyl acetate extract fraction III (E2-III) group	Received 60 mg/kg STZ and, after three days, received 100 mg/kg (E2-III) fraction orally via gastric gavage daily for 30 days
**S-7**	*C calcitrapa* ethyl acetate extract fraction IV (E2-IV) group	Received 60 mg/kg STZ and, after three days, received 100 mg/kg (E2-IV) fraction orally via gastric gavage daily for 30 days
**S-8**	*C calcitrapa* ethyl acetate extract fraction V (E2-V) group	Received 60 mg/kg STZ and, after three days, received 100 mg/kg (E2-V) fraction orally via gastric gavage daily for 30 days
**S-9**	*C calcitrapa* ethyl acetate extract fraction VI (E2-VI) group	Received 60 mg/kg STZ and, after three days, received 100 mg/kg (E2-VI) fraction orally via gastric gavage daily for 30 days
**S-10**	*C calcitrapa* ethyl acetate extract fraction VII (E2-VII) group	Received 60 mg/kg STZ and, after three days, received 100 mg/kg (E2-VII) fraction orally via gastric gavage daily for 30 days
**S-11**	*C calcitrapa* ethyl acetate extract fraction VIII (E2-VIII) group	Received 60 mg/kg STZ and, after three days, received 100 mg/kg (E2-VIII) fraction orally via gastric gavage daily for 30 days

## Data Availability

The data presented in this study are openly available in [Zenodo] at [https://doi.org/10.5281/zenodo.14983246] (date: 1 February 2025), reference number [14983246].

## Supplementary Materials

The following supporting information can be downloaded at: https://www.mdpi.com/article/10.3390/molecules30112394/s1, Figures S1–S5, which contain data about the Calibration curve (Figure S1), the standards of nepetin (Figure S2), the standards of kaempferide (Figure S3), the standards of Luteolin (Figure S4), and the plant material extraction procedure (Figure S5).

## Abbreviations

The following abbreviations are used in this manuscript: Diabetes mellitus (DM), Type 2 diabetes mellitus (T2DM), methanol (MeOH), Alkaline Phosphatase (ALP), aspartate aminotransferase (AST), alanine aminotransferase (ALT), superoxide dismutase (SOD), Malondialdehyde (MDA), thin-layer chromatography (TLC), Fourier Transform Infrared Spectroscopy (FTIR), high-performance liquid chromatography (HPLC), Mass spectrophotometry (MS), streptozotocin (STZ), enzyme-linked immunosorbent assay (ELISA), fasting blood glucose (FBG), genetic optimization for ligand docking (GOLD).
