# Supplementary material for: Bioassay-Guided Isolation and Active Compounds Identification of the AntiDiabetic Fractions of Centaurea calcitrapa Extract and the Predicted Interaction Mechanism
Source: Molecules. 2025 May 30;30(11):2394. doi: 10.3390/molecules30112394 (PMC12156352; doi:10.3390/molecules30112394)
Supplement: Supplementary file 1 [file molecules-30-02394-s001.zip › molecules-3573273-supplementary.pdf]

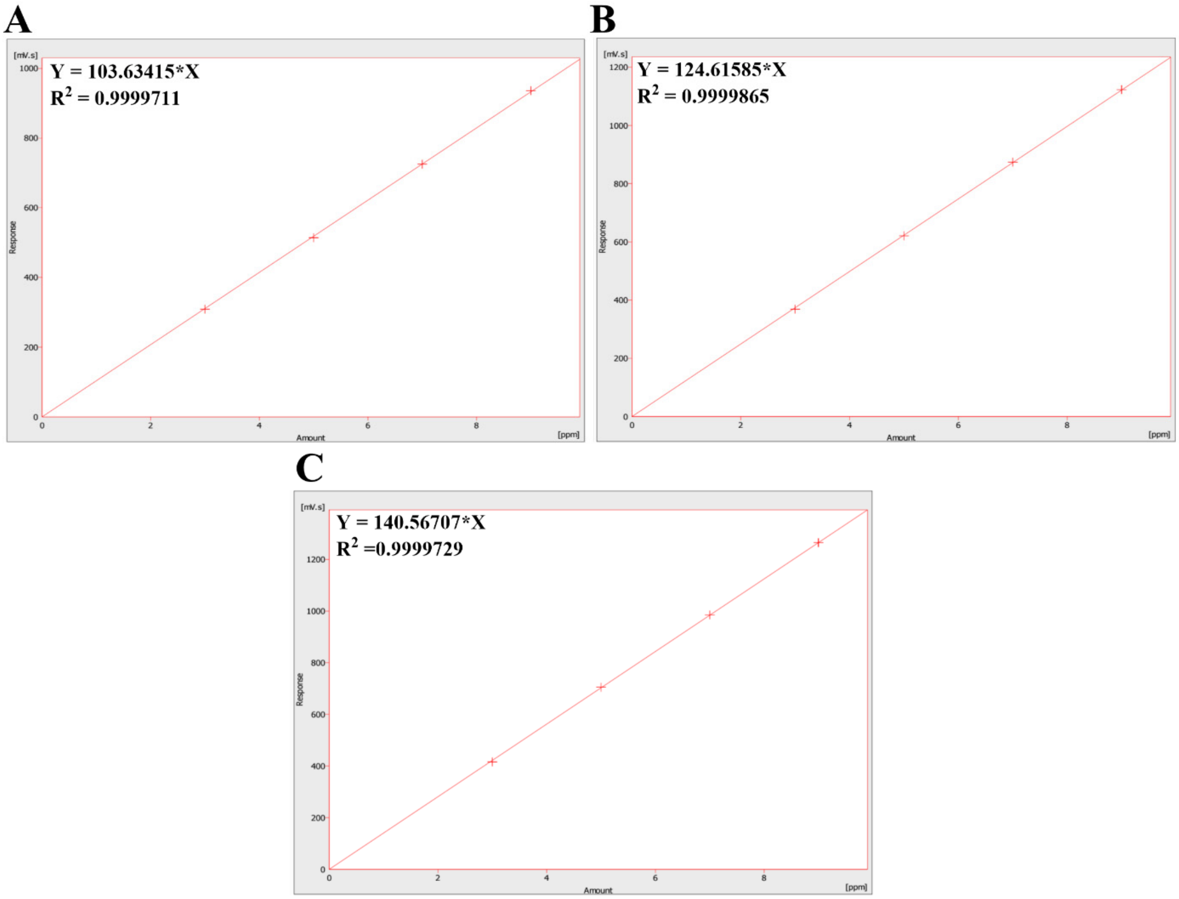

Figure S1: Calibration curve of (A) Nepetin, (B) kaempferide, (C) luteolin; in phosphate buffer pH 7.4

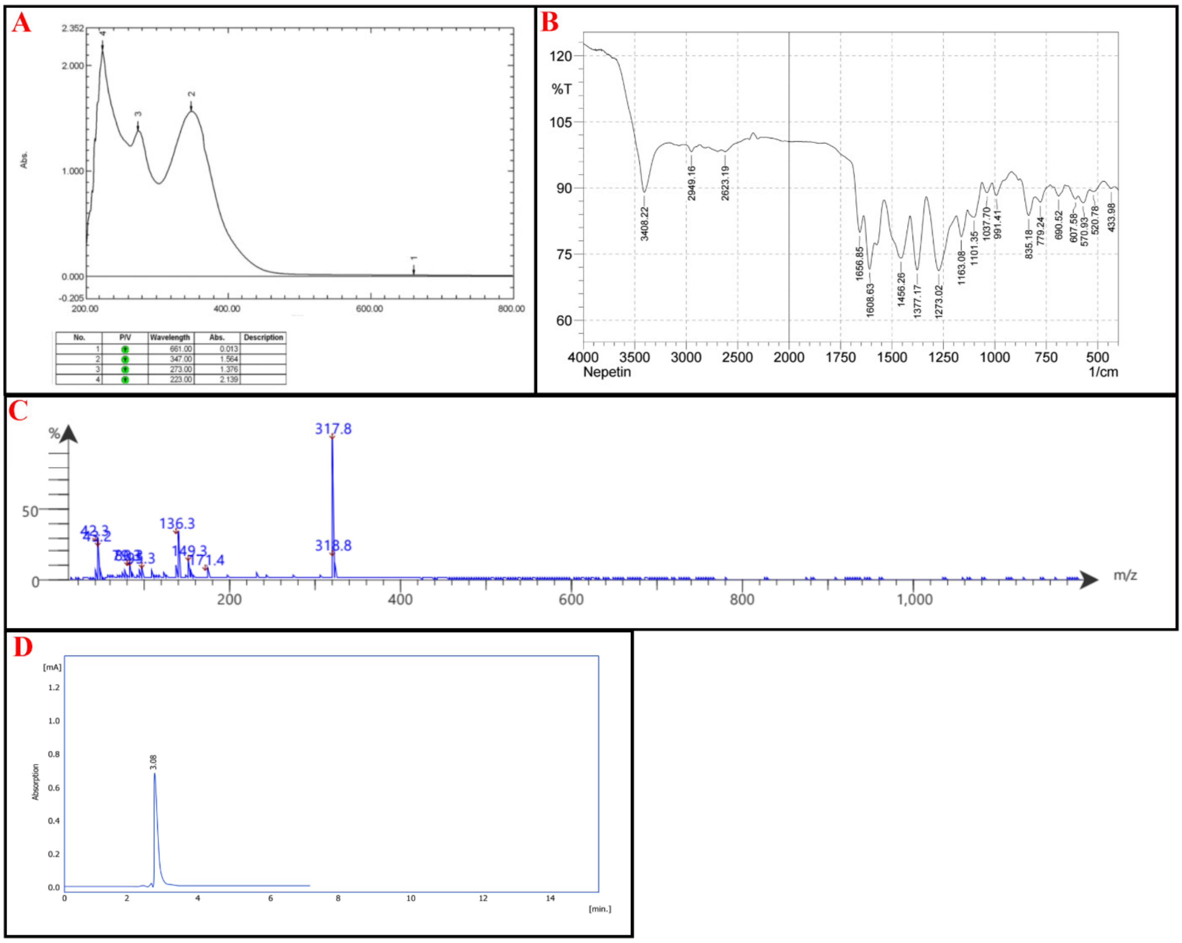

Figure S2: Nepetin compound standards, A) UV spectrophotometry, B) FT-IR, C) MS-spectrometry, D) HPLC, E) chemical structure.

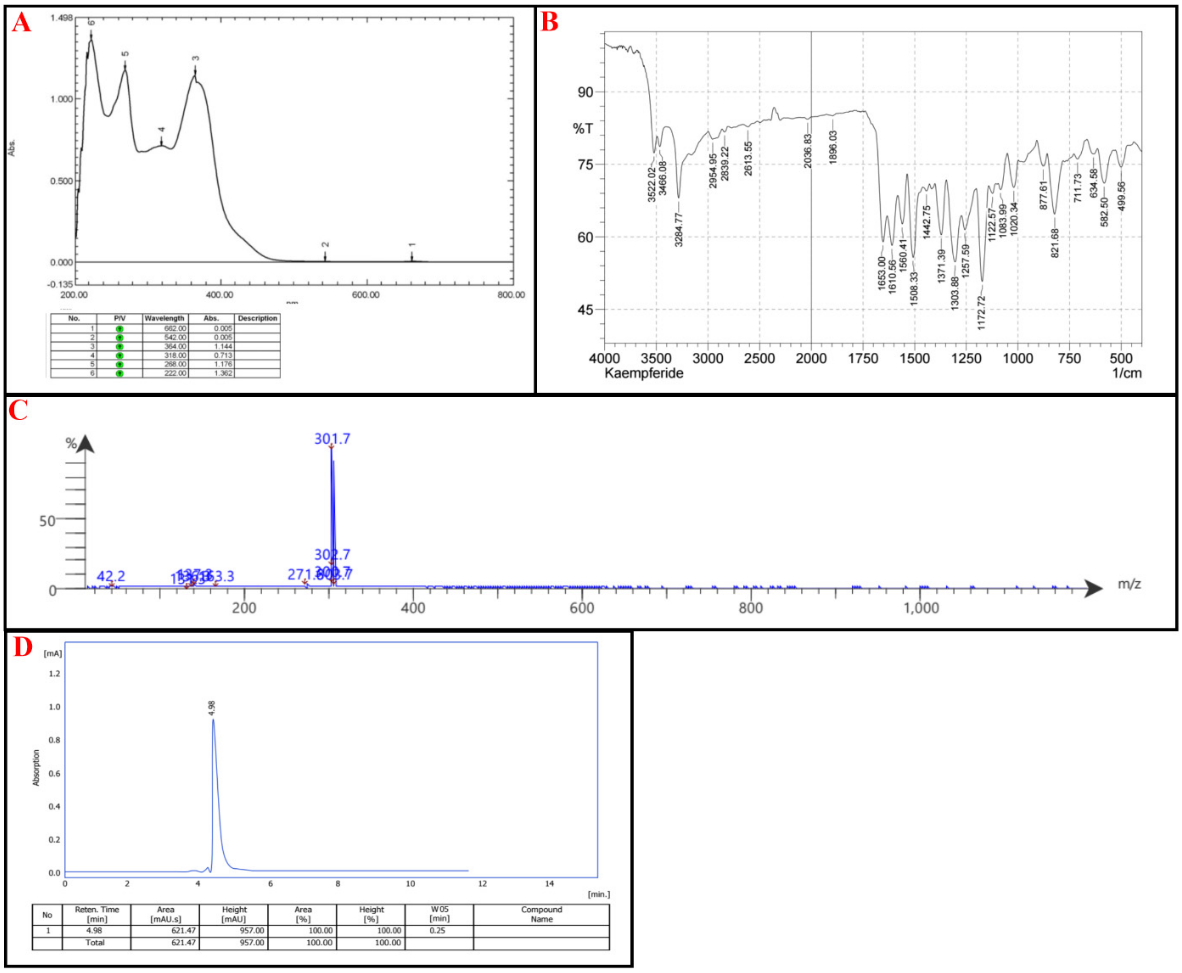

No.	P/V	Wavelength	Abs.	Description
1		662.00	0.005	
2		542.00	0.005	
3		364.00	1.144	
4		318.00	0.713	
5		268.00	1.176	
6		222.00	1.362	

No	Reten. Time [min]	Area [mAU.s]	Height [mAU]	Area [%]	Height [%]	W 05 [min]	Compound Name
1	4.98	621.47	957.00	100.00	100.00	0.25	
	Total	621.47	957.00	100.00	100.00		

Figure S3: Kaempferide compound standards, A) UV spectrophotometry, B) FT-IR, C) MS-spectrometry, D) HPLC, E) chemical structure.

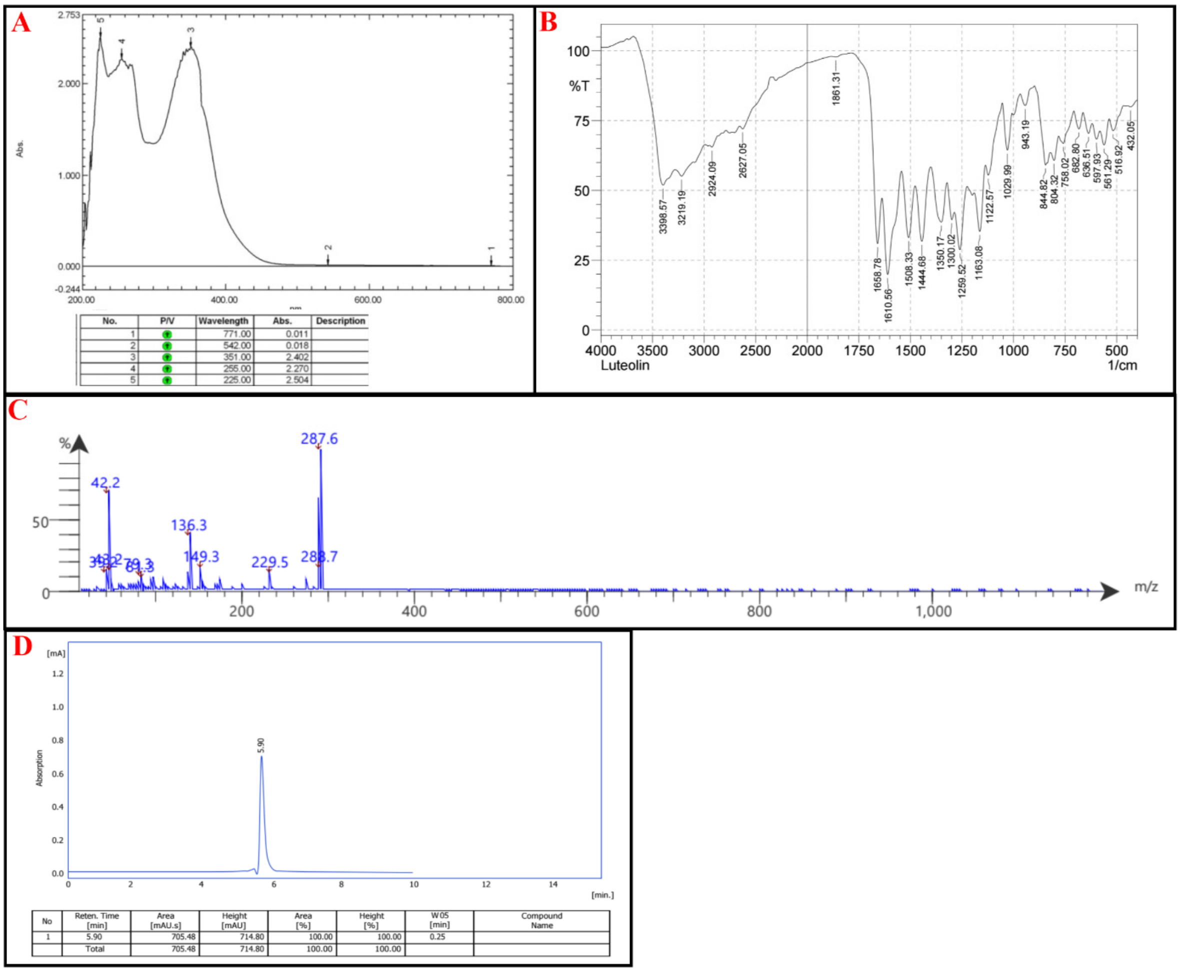

No.	P/V	Wavelength	Abs.	Description
1		771.00	0.011	
2		542.00	0.018	
3		351.00	2.402	
4		255.00	2.270	
5		225.00	2.504	

No	Reten. Time [min]	Area [mAU.s]	Height [mAU]	Area [%]	Height [%]	W05 [min]	Compound Name
1	5.90	705.48	714.80	100.00	100.00	0.25	
	Total	705.48	714.80	100.00	100.00		

Figure S4: Luteolin compound standards, A) UV spectrophotometry, B) FT-IR, C) MS-spectrometry, D) HPLC, E) chemical structure.

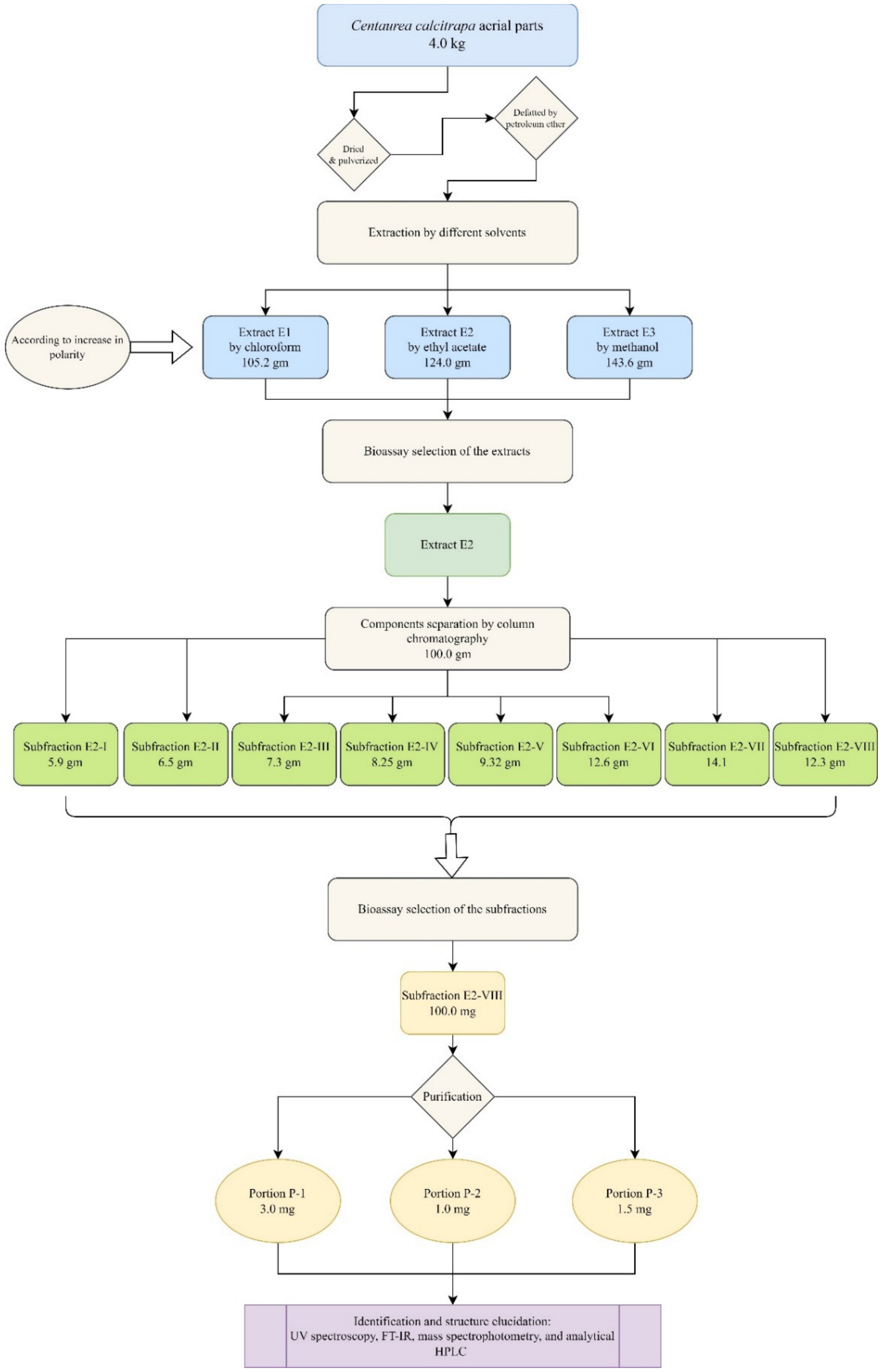

Figure S5: The plant material extraction procedure, including column chromatography, chromatographic separation, and purification
